# The mirrored cationic peptide as miRNA vehicle for efficient lung cancer therapy

**DOI:** 10.1002/mco2.273

**Published:** 2023-07-28

**Authors:** Wenyan Xu, Lingran Du, Lina Yu, Huiyu Cen, Fangyu Lin, Siran Wang, Zhixiong Ruan, Zhongxiao Lin, Xin Zhang, Na Zhou, Jishuo Chang, Xiyong Yu, Lingmin Zhang, Lu Liang

**Affiliations:** ^1^ Guangzhou Municipal and Guangdong Provincial Key Laboratory of Molecular Target & Clinical Pharmacology, The State & NMPA Key Laboratory of Respiratory Disease, School of Pharmaceutical Sciences & The Fifth Affiliated Hospital Guangzhou Medical University Guangzhou PR China; ^2^ Department of Preventive Dentistry, Affiliated Stomatology Hospital of Guangzhou Medical University, Guangdong Engineering Research Center of Oral Restoration and Reconstruction Guangzhou Key Laboratory of Basic and Applied Research of Oral Regenerative Medicine Guangzhou PR China; ^3^ Department of Ophthalmology Emory University Atlanta Georgia USA; ^4^ State Key Laboratory of Quality Research in Chinese Medicine Macau University of Science and Technology Avenida Wailong Taipa Macau PR China

**Keywords:** lung cancer, microRNA, MMP2‐sensitive peptide, RGD

## Abstract

Gene therapy has emerged as a potential approach for lung cancer therapy. However, the application of gene therapy is still limited by their properties, such as low specificity to the cancer cells, negatively charged groups, short systemic circulation time, and rapid degradation by nucleases. The progression of lung adenocarcinoma (LUAD) can be promoted through the methylation process of miR‐148a‐3p promoter, as confirmed by our previous research. In the current study, we are the first to design a mirrored Arg‐Gly‐Asp (RGD)‐modified cationic peptide (RD24) as a microRNA (miRNA) vehicle, which enabled to pack the miRNA (miR‐148a‐3p) efficiently and generate RD24/miR‐148a‐3p nanoparticles (RPRIN) by self‐assembling. RPRIN exhibited a high transfection efficiency in lung cancer cells via the conjugation between RGD and integrins on the surface of lung cancer cells. Furthermore, RD24 showed matrix metallopeptidase 2 (MMP2) responsiveness, which improved lung cancer cell inhibition induced by the miRNA intracellularly. In addition, RPRIN exhibits several advantages, such as prolonged circulation duration, reduced toxicity, and immune escape. Experiments conducted both in vitro and in vivo revealed that RPRIN effectively suppressed the growth and progression of lung cancer. Thus, the mirrored RGD‐modified cationic peptide showed great potential in transducing miRNA for lung cancer therapy.

## Graphical abstract

We developed a mirrored RGD‐modified cationic peptide (RD24) as a miRNA vehicle. RD24 enabled to encapsulate miR‐148a‐3p and generated RD24/miR‐148a‐3p (RPRIN), which effectively transduced miRNA into lung cancer cells. Moreover, RPRIN showed extended circulation time, lower toxicity, and specificity to lung cancer cells. The mirrored RGD‐modified cationic peptide may show great potential in miRNA delivery for lung cancer therapy.

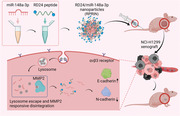

## 1 INTRODUCTION

Lung cancer is one of the severest carcinomas that affect human health due to high morbidity and mortality. Traditional therapies, such as surgical resection, chemotherapy, and radiotherapy, have been applied to eliminate lung cancer. Nonetheless, these standard treatments exhibit restricted therapeutic efficacy and notable adverse effects on patients. To solve the current issues, it is urgent to develop novel strategies for efficient and safe lung cancer therapy.

MicroRNAs (miRNAs), small non‐coding RNA molecules, are discussed as the fundamental regulators for gene expression.^1^ Growing reports have demonstrated the involvement of miRNA in the development and progression of tumors.^2^, ^3^ Additionally, several studies have identified varying levels of miRNAs in normal and tumor tissues. miRNAs are considered the diagnostic markers of cancer, which is a potential target of future applications.^4^, ^5^, ^6^ Growing studies have indicated that miR‐148a locus encoding two miRNAs, miR‐148a‐3p/5p, are abnormally expressed in different types of tumors, and associated with tumor size, TNM stage, metastasis, and prognosis.^7^ Our findings indicate that miR‐148a‐3p, which is regulated by promoter methylation, inhibits the progression of lung adenocarcinoma (LUAD).^8^ Thus, miR‐148a‐3p may potentially serve as an effective tumor marker for the treatment of lung cancer.

Despite years of studying miRNAs in lung cancer, they have not yet reached the stage of clinical application. Naked miRNAs uptake by cells is limited by negatively charged groups,^9^ limited systemic circulation time,^10^, ^11^ undesired off‐target effects,^12^ and rapid degradation by nucleases.^13^, ^14^ To solve these issues, the application of gene vehicles to deliver miRNAs is attracted by their performance to protect the loaded miRNAs from the external factors, thereby prolonging circulation time, inhibiting enzymatical degradation, and specified accumulation.^15^, ^16^, ^17^, ^18^

Gene vehicle‐mediated miRNA delivery has been studied in cancer treatment, immunotherapy, tissue engineering (TE), and neurodegenerative disorders. But the vehicles are still not satisfying in lung cancer therapy.^19^ Based on the enhanced permeability and retention (EPR) effect, many nanomedicines could passively accumulate in tumor sites.^20^ However, the nanoparticles (NPs) without specificity compromised the therapeutic effects of lung cancer.^21^, ^22^ Thus, it is in great demand to develop a novel style of gene vehicles that can promote miRNA delivery.

**FIGURE 1:**
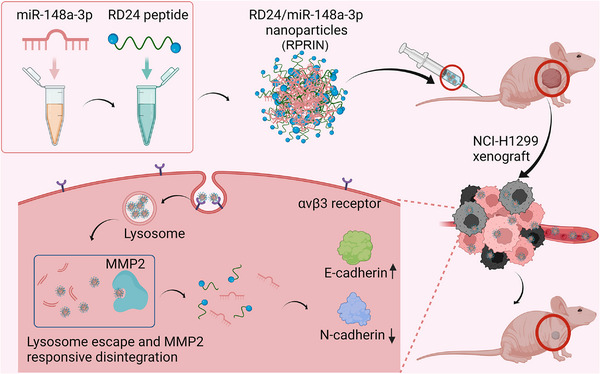
Schematic diagram of the preparation of RPRIN and its mechanism on the inhibition of lung cancer cells. RPRIN: RD24/miR‐148a‐3p. The schematic diagram was created with BioRender.com.

The specific ligands to target the receptors are essential to improve the selectivity toward receptors on the cell surface. NPs possess the homing element, such as an antibody or peptide that specifically binds to the target tissue, improving the therapeutic effects.^23^ The functional peptides have become useful tools in cancer therapy. Integrin receptors, including αvβ3, are highly overexpressed in tumors, and Arg‐Gly‐Asp (RGD) acts as a specific ligand to recognize the integrin sites and enhance the tumor targeting.^24^, ^25^ RGD‐modified NPs have shown great potential in delivering anticancer drugs or contrast agents for the diagnosis and treatment of cancer, which was confirmed to enter specific target cells via receptor‐mediated endocytosis.^26^, ^27^ The smart peptides with environmental responsiveness to the intracellular environment facilitated the therapeutic effect.^28^ Matrix metalloproteinase 2 (MMP2) is overexpressed in many tumors, and high MMP2 levels are associated with tumor metastasis and progression.^29^ In previous studies, the multifunctional nanocarriers with MMP2 responsiveness were designed, which were degradable in the presence of MMP2.^30^, ^31^, ^32^ In our study, MMP2‐responsive nanoparticles were constructed using the Pro‐Leu‐Gly‐Leu‐Ala‐Gly (PLGLAG) peptide, which can be cleaved by MMP2. And MMP2‐responsive nanoparticles were sensitive to MMP2 and improve drug release. As shown in Figure 1, for the first time, we designed a cationic peptide with a mirrored RGD modification, featuring four arginine residues at both ends of the MMP2‐cleavable peptide PLGLAG (RGDGSRRRRPLGLAGRRRRGSRGD, RD24), which was used to carry the lung cancer suppressive miRNA, miR‐148a‐3p. We evaluated the loading efficiency and the transfection efficiency of RD24 on miRNA. Furthermore, lung cancer cell inhibition was performed both in vitro and in vivo. The novel nanodelivery system RD24 we constructed may exhibit great potential in miRNA delivery, which can deliver miR‐148a‐3p to target and penetrate into the tumor tissues, improve endosomal escape, and release therapeutic miRNA into the cytoplasm to silence target gene for lung cancer therapy.

## 2 RESULTS

### 2.1 Preparation and characterizations of the nanoparticles

Table S1 presents the detailed sequences and abbreviations of the nanoparticles that we developed by incorporating miR‐148a‐3p into a series of peptides. These peptides contain MMP‐2‐recognizable sequences PLGLAG, with or without RGD sequences at both ends of the PLGLAG sequences. We detected the cytotoxicity of these peptide sequences by Cell Counting Kit‐8 (CCK‐8) assay. RR14, RR19, and RD24 showed no significant cytotoxicity in the cell lines, such as A549 (lung adenocarcinoma cells), NCI‐H1299 (lung adenocarcinoma cells), or BEAS‐2B cells (human bronchial epithelial cells) (Figure S1). These peptides showed no significant cytotoxicity to the tested cell lines such as A549, NCI‐H1299, and BEAS‐2B cells, implying the safety in the potential applications for miRNA delivery.

**FIGURE 2:**
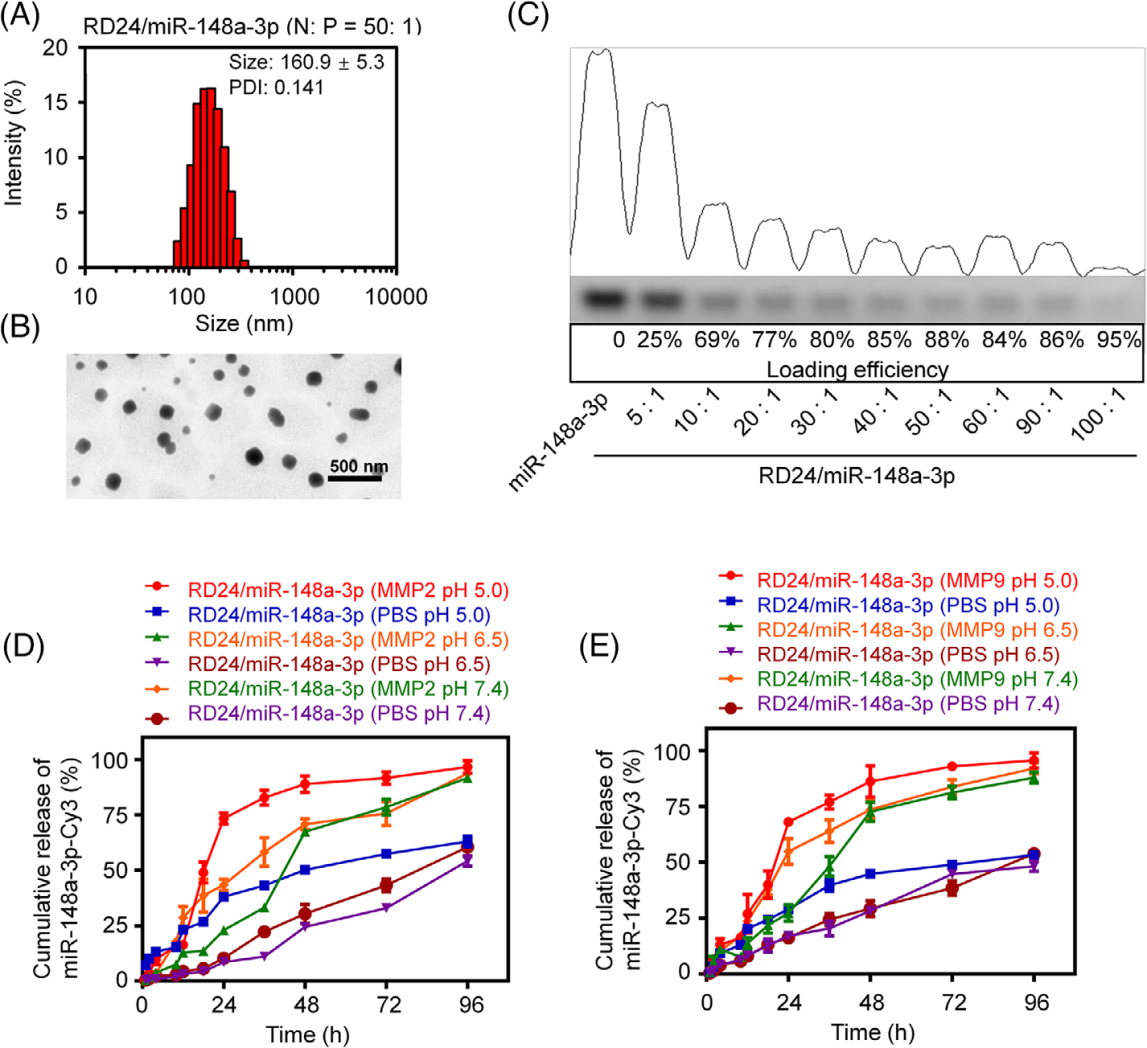
The properties of RD24/miR‐148a‐3p NPs (RPRIN). RPRIN was prepared by self‐assembling, then (A) the size distributions were detected by Zeta Sizer, and (B) the morphological characteristics were analyzed by TEM. (C) The loading efficiency of RD24 to miR‐148a‐3p. The cumulative releases of miR‐148a‐3p from RPRIN were quantitatively evaluated in (D) MMP2 or (E) MMP9 medium with different pH values at the indicated time points.

Both the Zeta Sizer and the transmission electron microscope (TEM) analysis revealed that RR14/miR‐148a‐3p (PIN), RR19/miR‐148a‐3p (RPIN), and RD24/miR‐148a‐3p (RPRIN) formed uniform nanoparticles at 50:1 (w/w) (Figure 2A,B and Figure S2A–D). Therefore, the weight ratio of *N*/*P* = 50:1 was used in the following experiments. All of the nanoparticles we developed had a size ranging from 150 to 250 nm, with a polydispersity index (PDI) of less than 0.5 (Figure 2A,B and Figure S2A–D). Meanwhile, we measured the loading efficiency at different weight ratios (5, 10, 20, 30, 40, 50, 60, 90, and 100) of RR14, RR19, and RD24 to miR‐148a‐3p, respectively. The agarose gel electrophoresis results showed that the loading efficiencies of miRNA with RR14, RR19, and RD24 were all more than 80% when the weight ratios were over 40. And the loading efficiencies of RR14, RR19, and RD24 to miR‐148a‐3p were 92%, 83%, and 88% at 50:1 (w/w), respectively (Figure 2C and Figure S2E,F). The zeta potential of all the prepared nanoparticles was ranging 10−15 mV (Figure S3A). The DLS analysis showed that the size and PDI of PIN, RPIN, and RPRIN were extremely stable in serum within 5 days, which was beneficial to improve the circulation lifetime (Figure S3B–D)

MMP2 and MMP9 are overexpressed in many tumors, and high MMP2 and MMP9 levels promote tumor metastasis and progression.^29^ Our results confirmed that MMP2 and MMP9 were highly expressed in NCI‐H1299 cells but not in normal cell line BEAS‐2B (Figure S4). We found that the release rates of RD24/miR‐148a‐3p NPs were rapidly increased since 12 h, and more than 80% of Cy3‐miR‐148a‐3p were released within 48 h in the presence of 50 ng/mL MMP2 or MMP9 in pH 5.0 (Figure 2D,E). Interestingly, in the lower pH values, the higher cumulative releases of miR‐148a‐3p were observed within 48‐h incubation in the MMP2 or MMP9 medium (Figure 2D,E).

The mirrored RGD‐modified cationic peptide, RD24, enabled to complex miRNA, which formed uniform nanoparticles and showed a high loading efficiency. The miRNA‐containing nanoparticles were acidic, MMP2 and MMP9 sensitive, providing the miRNA with environmental responsiveness.

### 2.2 Cellular uptake and lysosome escape assay

The confocal laser scanning microscope (CLSM) and flow cytometer (FCM) was used to analyze the cellular uptake of RPRIN. Cy3 conjugated with miR‐148a‐3p was used as the fluorescence marker. Both CLSM and FCM analyses indicated that Cy3‐miR‐148a‐3p equivalent to 4 μg/mL exhibited the maximum cellular uptake (with Cy3‐positive cells more than 97%) (Figure S5A). Moreover, we found that the maximum efficiency of miRNA uptake (>99%) was observed after incubating with RPRIN‐Cy3 NPs for 8 h (Figure S5B). Further extension of incubation time did not improve the transfection efficiency significantly. Thus, RPRIN with miRNA‐148a‐3p at the dosage of 4 μg/mL Cy3‐miR‐148a‐3p and 8‐h incubation was optimized for further studies.

**FIGURE 3:**
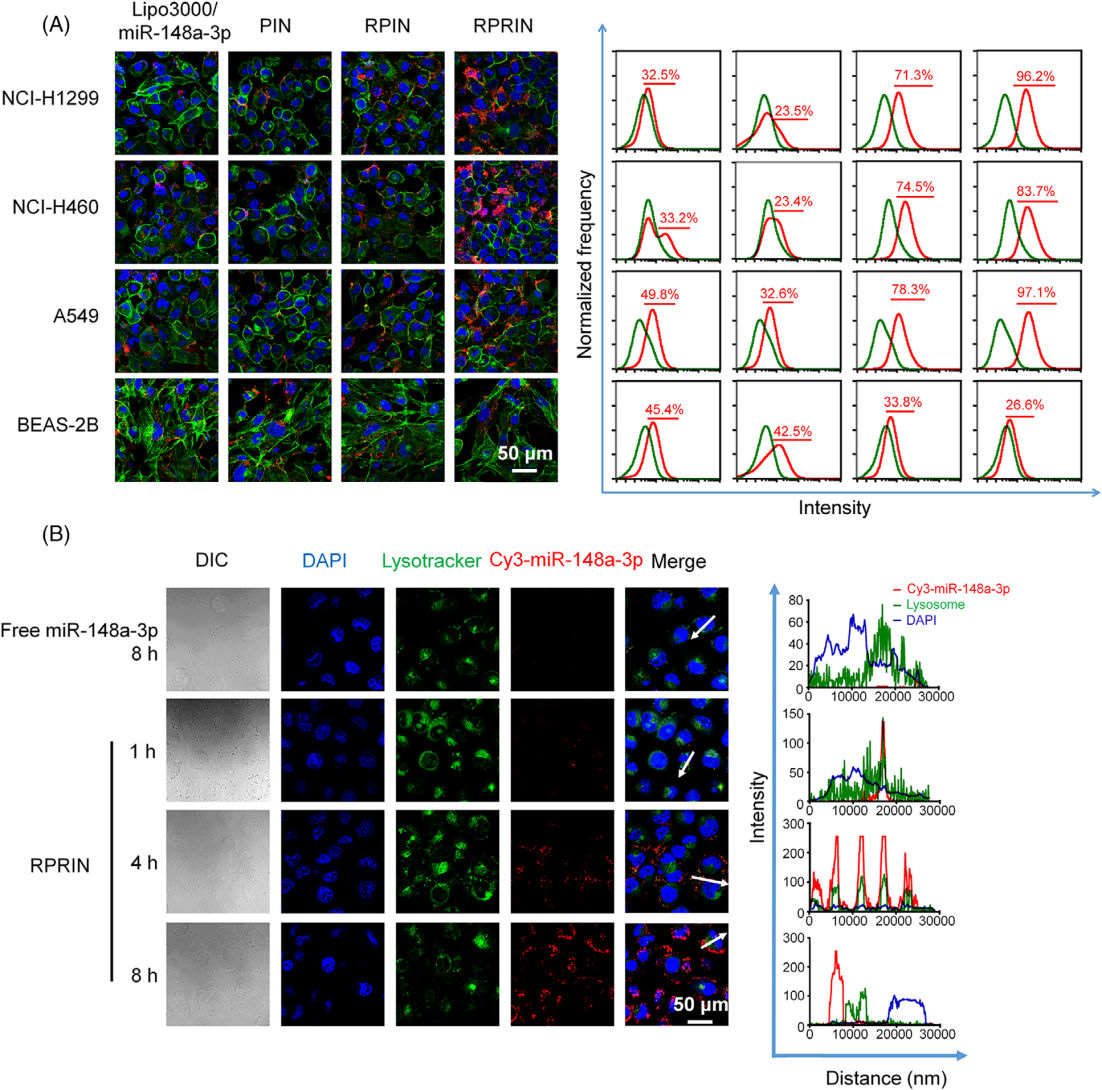
The cellular uptake in different conditions and lysosome escape. (A) The cellular uptake of different formulations (Lipo3000/miR‐148a‐3p, PIN, RPIN, and RPRIN) containing 4 μg/mL miR‐148a‐3p in different cell lines (NCI‐H1299, NCI‐H460, A549, and BEAS‐2B). Blue: DAPI; green: FITC palloidin; red: Cy3‐miR‐148a‐3p. (B) The lysosome escapes of Cy3‐miR‐148a‐3p. NCI‐H1299 cells were incubated with RPRIN for 1, 4, and 8 h, respectively. The fluorescence intensities of DAPI, Cy3‐miR‐148a‐3p, and lysotracker were quantitatively analyzed based on the white arrows. The distance of the white arrow corresponded to the value on the *x*‐axis. Excitation/emission: FITC‐Phalloidin (488 nm/525 nm); Cy3 (555 nm/590 nm); DAPI (405 nm/461 nm).

We evaluated the cellular uptake of PIN, RPIN, and RPRIN in different cell lines. The lung cancer cell lines NCI‐H460, NCI‐H1299, and A549 exhibited stronger fluorescence intensities than human normal lung epithelial cells BEAS‐2B (Figure 3A and Figure S6A–D) after the treatment with RPIN and RPRIN, and RPRIN showed the most effective cellular uptake of miR‐148a‐3p (Figure 3A and Figure S6A–D). Interestingly, we found that the cellular uptake treated with PIN did not show a significant difference in these cell lines, implying that the nanoparticles without RGD showed no specificity to the cells.

The αvβ3 receptor inhibitor was used to reveal the mechanism of RGD‐induced cellular uptake. The LSCM results showed that cellular uptake of RPRIN was significantly decreased when the NCI‐H1299 cells were pretreated with Cilengitide TFA. However, it had no significant change in BEAS‐2B cells pretreated with or without Cilengitide TFA (Figure S7). These results indicated that RPRIN was internalized by tumor cells mediated by RGD‐specific receptor αvβ3. These results indicated that RPRIN enabled to target the lung cancer cells effectively.

CLSM was used to evaluate the lysosome escape induced by RPRIN. After being labeled with lysotracker, the lysosomes emitted green fluorescence, while RPRIN was labeled with Cy3 and emitted red fluorescence. When the green and red fluorescence were merged, the resulting yellow fluorescence was observed. CLSM analysis showed that yellow fluorescence appeared after incubating NCI‐H1299 cells with RPRIN for 1 h. Moreover, the stronger yellow fluorescence intensity was still observed within 4 h (Figure 3B). These results indicated that quite a few of NPs were captured by lysosomes after cellular uptake. Interestingly, the miRNA escaped from the lysosomes after 8 h, evidenced by the green fluorescence separating from the red one. These results demonstrated that sufficient cellular uptake and efficient lysosome escape was achieved by the loading with RD24, which were essential for the following lung cancer therapy.

### 2.3 The inhibition of lung cancer cells in vitro

**FIGURE 4:**
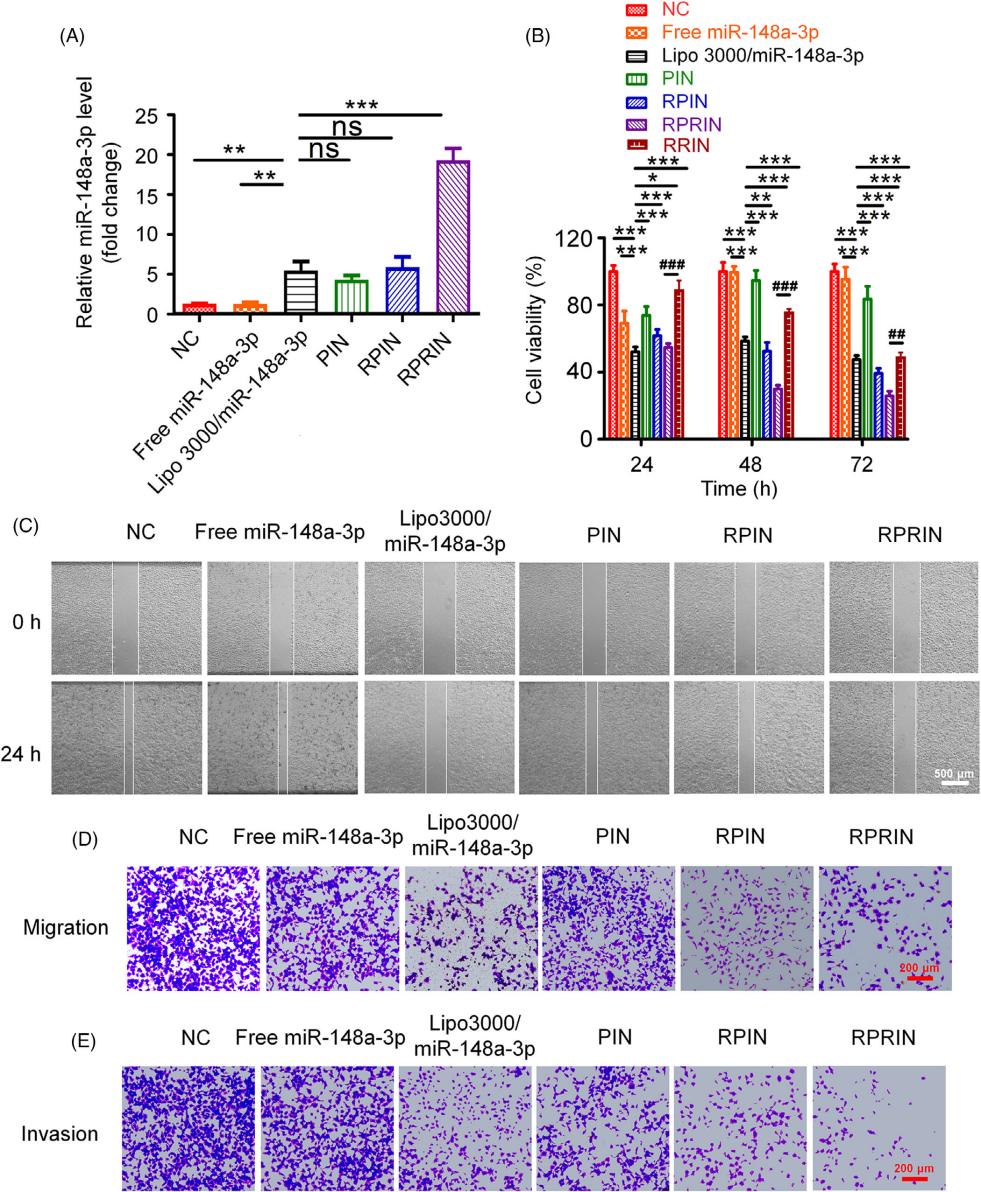
The effect of nanoparticles on cell proliferation, migration, and invasion. (A) RT‐qPCR was used to detect the mRNA levels of miR‐148a‐3p in NCI‐H1299 cells 6 h after treatment with free miR‐148a‐3p, Lipo3000/miR‐148a‐3p, PIN, RPIN, and RPRIN, respectively. (B) The viabilities of NCI‐H1299 cells at 24, 48, and 72 h after indicated treatment were measured by CCK8, respectively. (C) Cell migration was measured by wound healing assay after the indicated treatment. Scale bar: 500 μm. Cell migration (D) and invasion (E) were measured by transwell assay. Scale bar: 200 μm. *p < 0.05; **p < 0.01; ***p < 0.001.

We further evaluated the antitumor effects induced by the nanoparticles on lung cancer cells in vitro. RT‐qPCR detection suggested that PIN and RPIN (miR‐148a‐3p equivalent to 4 μg/mL) exhibited considerable transfection efficiency compared to the transfection reagent Lipo3000. Moreover, RPRIN showed a significantly higher transfection efficiency than the commercial transfection reagent Lipo3000 at 6 h after transfection (Figure 4A). Then, we evaluated the inhibition effect induced by nanoparticles toward NCI‐H1299 cells by CCK8 assay, which indicated that RPIN and RPRIN significantly inhibited the proliferation of NCI‐H1299 cells in a time‐dependent manner, with the inhibitory rates of approximately 60% and 70% at 72 h, respectively (Figure 4B). Subsequently, we analyzed the role of nanoparticles in cell migration and invasion of lung cancer cells by wound‐healing assay, transwell migration, and invasion assay. We found that RIPIN significantly suppressed cell migration, which exhibited a more noticeable effect than transfection reagent Lipo3000 (Figure 4C,D and Figure S8A,B). For transwell invasion assay, RPIN and RPRIN significantly inhibited cell invasion with coverage rates at approximately 20% and 10% on the lower surface (Figure 4E and Figure S8C). The above results reveal that RPRIN mediates effective cancer cell inhibition, migration, and invasion, indicating the antitumor effects of RPRIN in vitro. The results above demonstrated that RPRIN induced effective cancer cell inhibition.

### 2.4 In vivo tracking and circulating lifetime

**FIGURE 5:**
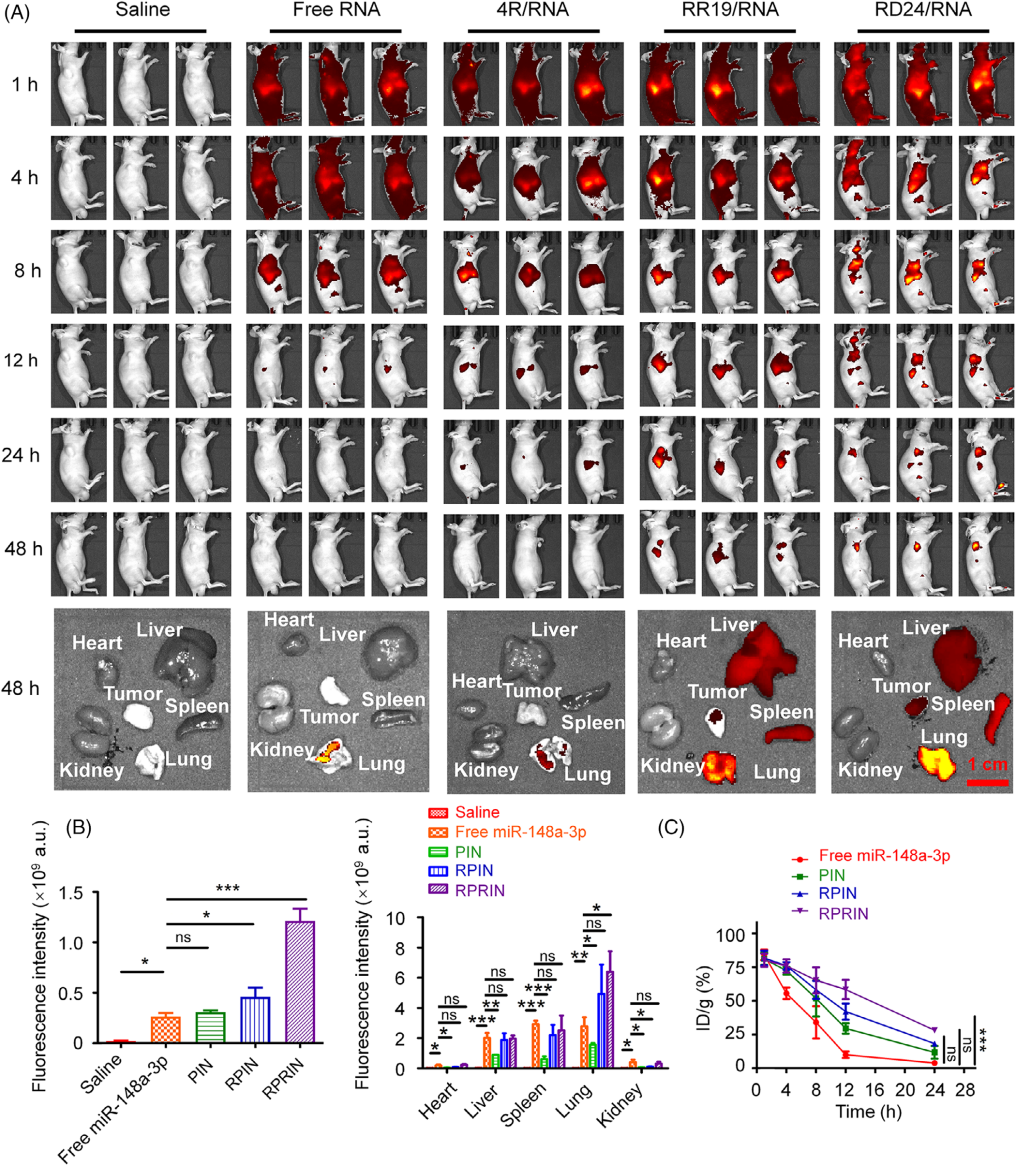
Biodistribution and circulation of different nanoparticles in vivo. The NCI‐H1299 xenograft tumor‐bearing BALB/c nude mice were administrated with saline, free Cy3‐miR‐148a‐3p, PIN, RPIN, and RPRIN (equivalent to 20 μg Cy3‐miR‐148a‐3p per mouse) by intravenous injection from the tail vein, respectively. IVIS Lumina III was applied for the detection of the distribution of nanoparticles at 1, 4, 8, 12, 24, and 48 h after injections. (A) Biodistribution of nanoparticles in vivo and ex vivo images of the major organs. (B) The quantitative analysis of the fluorescence of the major organs and tumors. (C) Circulation lifetime of nanoparticles was detected at 1, 4, 8, 12, and 24 h after administration. *p < 0.05; **p < 0.01; ***p < 0.001.

The NCI‐H1299 xenograft tumor‐bearing BALB/c nude mice were administrated with saline, free miR‐148a‐Cy3, PIN, RPIN, and RPRIN (equivalent to 20 μg Cy3‐miR‐148a‐3p per mouse) by intravenous injection from the tail vein for the detections of in vivo distribution and circulation lifetime, respectively. As shown in Figure 5, after the administration for 1 h, the systemic distribution of different formulations in mice was observed in free Cy3‐miR‐148a‐3p‐, PIN‐, RPIN‐, and RPRIN‐treated groups. After 12 h, the Cy3‐miR‐148a‐3p‐loaded nanoparticles exhibited higher accumulation in tumor sites than free Cy3‐miR‐148a‐3p. After 48 h, a much stronger fluorescence signal was captured in RPRIN‐treated mice than in RPIN or PIN, indicating that the mirrored RGD at the two ends of the RR14 sequence promoted the accumulation and retention of miR‐148a‐3p in tumor tissues. The consistent results were obtained from ex vivo imaging of the main organs and tumors, in which Cy3‐miR‐148a‐3p‐loaded nanoparticles were significantly accumulated in tumors, especially for RPRIN‐treated mice. Interestingly, no fluorescence signals were detected in free miR‐148a‐3p treated mice at 24 and 48 h (Figure 5A,B). These results indicated that naked miRNA was metabolized quickly.

We also evaluated the circulation lifetime in the BALB/c nude mice. The whole‐blood samples at different time points were collected to detect the fluorescence signals. Approximately 90% of free miR‐148a‐3p without any protection was cleared within 12 h after injection. After the administration for 24 h, the blood retention rates of PIN, RPIN, and RPRIN were about 10%, 18%, and 28%, respectively, while almost all the free Cy3‐miR‐148a‐3p was cleared. We found that the Cy3‐miR‐148a‐3p combined with RR14, RR19, and RD24 showed a longer circulation lifetime than free Cy3‐miR‐148a‐3p (Figure 5C). The extended circulation lifetime may increase the possibility of tumor‐specific delivery.

The in vivo tracking and ex vivo imaging confirmed the effective tumor accumulation induced by RPRIN, which demonstrated that the dual modification of RGD at both ends of the peptides was useful for the tumor‐specific delivery.

### 2.5 In vivo tumor xenograft assay

**FIGURE 6:**
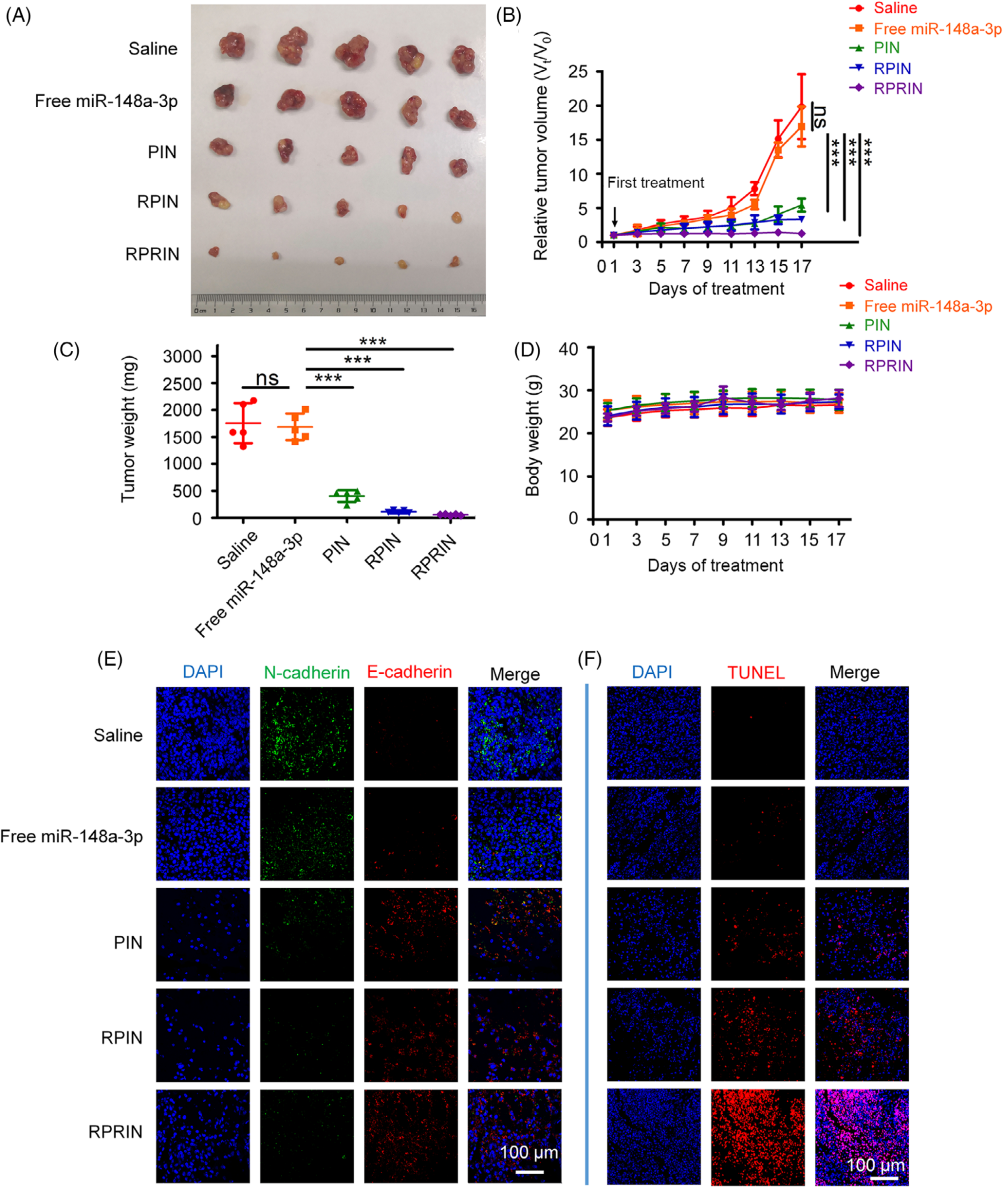
The effect of different formulations on tumor growth in vivo (*n* = 5). The BALB/c nude mice bearing NCI‐H1299 tumors were treated with saline, free miR‐148a‐3p, PIN, RPIN, and RPRIN by intravenous injection through the tail vein every 2 days for 2 weeks. The tumor volumes and the bodyweights of mice were measured before each treatment. Relative tumor volume (RTV)  =  *V*_t_/*V*_0_, where *V*_0_ is the tumor volume at day 0 of treatment and *V*_t_ is the tumor volume at day *t* of treatment. (A) The excised tumors were imaged. (B) The changes in the tumor volumes were determined. (C) The excised tumors were weighed. (D) The changes of the bodyweights. (E) The expression of E‐cadherin and N‐cadherin in tumor tissues was detected by immunofluorescence. (F) TUNEL staining was used to detect the cell apoptosis in the tumor tissues. Scale bar: 100 μm. *p < 0.05; **p < 0.01; ***p < 0.001.

The antitumor effects of PIN, RPIN, and RPRIN were evaluated in the NCI‐H1299 cell tumor‐bearing BALB/c nude mice. The tumor volumes were measured before every injection, and the tumors were harvested and weighed after the therapeutic period. The saline and free miR‐148a‐3p‐treated groups were used as control. Our results indicated that PIN, RPIN, and RPRIN dramatically suppressed tumor growth in vivo by monitoring tumor volume and the size of extracted tumors (Figure 6A–C). The treatment with RPRIN exhibited the best antitumor effect than the PIN‐ and RPIN‐treated groups. No obvious changes were observed in the bodyweight after treatment with PIN, RPIN, and RPRIN, indicating that these nanoparticles exhibited neglectable toxic effects (Figure 6D). Moreover, the immunofluorescence staining results demonstrated that PIN, RPIN, and RPRIN restored the expression of E‐cadherin and inversely inhibited the expression of N‐cadherin (Figure 6E). The TUNEL staining indicated that a high level of apoptosis happened in the tumor tissues in RPRIN‐treated groups (Figure 6F). EdU assay was a common approach for the detection of DNA replication and cell proliferation. The EdU staining results revealed that higher ratios of EdU‐positive cells were obviously found in free miR‐148a‐3p‐, saline‐, PIN‐, and RPIN‐treated groups. However, the EdU‐positive cells were rarely observed in RPRIN groups (Figure S9). HE staining suggested that the administration of PIN, RPIN, and RPRIN showed no obvious side effects on major organs (Figure S10). These results indicate that using RD24 for miRNA delivery was an alternative strategy for the therapy of lung cancer.

## 3 DISCUSSION

In our earlier research, we have demonstrated that the reduced expression of miR‐148a‐3p in lung adenocarcinoma is linked to the methylation of DNA in CpG islands located within the promoter regions of the miRNA.^8^ Furthermore, we discovered that the target of miR‐148a‐3p is the mitogen activated protein (MAP) kinase kinase 9 (MAP3K9). The upregulation of miR‐148a‐3p or the silencing of MAP3K9 led to a noteworthy reduction in cell proliferation, migration, invasion, cytoskeletal recombination, and epithelial–mesenchymal transition. Thus, the decrease of miR‐148a‐3p expression associated with promoter methylation can induce lung cancer metastasis by regulating MAP3K9 expression. Based on our findings, it appears that there exists a strong correlation between the methylation of miR‐148a‐3p and tumor metastasis. Furthermore, decreased expression of miR‐148a‐3p is linked with worse overall survival in patients with LUAD. Thus, the methylation status of miR‐148a‐3p could serve as an effective prognostic/predictive marker in lung cancer cells.^8^ Although miR‐148a‐3p plays a potential role in lung cancer therapy, the application of miR‐148a‐3p is limited by the properties of nucleic acids, such as strongly negative charge and nuclease sensitivity.

To improve the cellular uptake of miR‐148a‐3p, miR‐148a‐3p‐loaded nanoparticles (PIN, RPIN, and RPRIN) were designed for treating lung cancer. Arginine (R) is a natural amino acid processing active binding site based on electrostatic and hydrogen‐bonding coordination. The electrostatic repulsion and steric repulsion of arginine‐rich peptides play a crucial role in encapsulating the nucleic acids and improve the efficacy of delivering miR‐148a‐3p to cells. Herein, we synthesized a mirrored RGD‐modified cationic peptide to facilitate the delivery of miRNA. The peptide was designed with four arginine residues at either end of the MMP2‐cleavable peptide PLGLAG (RRRR‐PLGLAG‐RRRR) and was modified with RGD to improve its targeting ability.

The major challenge in miRNA delivery is the effective delivery of miRNA antagonists or miRNA mimics to the tumor cells. The complex extracellular matrix (ECM) is involved in preventing miRNA from contacting cancer cells. Nontumor cells including tumor‐associated macrophages, neutrophils, and monocytes in the tumor microenvironment (TME) also act as the crucial extracellular barriers. These nontumor cells can uptake and capture miRNAs without specificity in the delivery system of miRNA.^33^ To improve the ability of tumor‐associated active targeting, RGD was used to combine one or two ends of RRRR‐PLGLAG‐RRRR. These polypeptides RR14 (RRRRPLGLAGRRRR), RR19 (RGDGSRRRRPLGLAGRRRR), and RD24 (RGDGSRRRRPLGLAGRRRRGSRGD) could encapsulate miR‐148a‐3p efficiently and generate PIN, RPIN, and RPRIN nanoparticles by self‐assembling.

Cellular uptake of RPRIN was in concentration‐dependent and time‐dependent manner. For the concentration‐dependent cellular uptake, when the concentration of RPRIN was low, almost all the RPRIN was ingested by cells. And with the increase of RPRIN concentration, the intracellular miR‐148a‐3p increased accordingly. However, the αvβ3 receptors on the surface of cell membrane were saturated by the RGD peptides of RPRIN in the concentration of 4 μg/mL, so the cellular uptake of RPRIN peaked. For the time‐dependent cellular uptake, the cellular uptake of RPRIN increased in a time‐dependent manner and the intracellular miR‐148a‐3p increased accordingly. Both RPRIN and miR‐148a‐3p would be degraded by cells over 8 h, so there was a dynamic balance between cellular uptake and degradation of RPRIN and miR‐148a‐3p at 8 h.

Although miR‐148a‐3p can successfully target tumor tissues, how to improve the uptake of miR‐148a‐3p is still challenging. Due to endocytosis process, most of the miRNAs are captured in the endosomes and are further degraded in the late endosomes/lysosomes during the intracellular delivery of miRNAs. It is necessary to develop effective strategies to improve endosomal escape and release therapeutic miRNA into the cytoplasm to silence target gene. In our study, RPRIN can be released into the cytoplasm in a pH‐dependent manner. RPRIN can be predigested at the TEM (pH 6.5–6.8), which makes nanoparticles become structurally loose. Then the nanoparticles were captured into the lysosomes (pH 4.5–5.5), they were further digested. Finally, miR‐148a‐3p escaped from the lysosomes after 8 h.

The nanoparticles achieve lysosome escape through proton sponge effect. When the pH in the lysosomes decreases, the lysosomes can capture a large number of protons and cause the internal flow of Cl^−^ and H_2_O, resulting in the osmotic swelling of the lysosome.^34^, ^35^ Finally, the lysosomes are broken and the captured formulations are released into the cytoplasm. In our study, after the positively charged nanoparticles enter into the cells and captured by the lysosomes, the unsaturated amino groups on the particles chelate protons provided by the proton pumps (V‐ATPase). A proton traps Cl^−^ and H_2_O in the lysosomes, causing swell and rupture of lysosomes, and the components are finally released into the cytoplasm.

Although the abundant MMP‐2 is present in the extracellular matrix within the tumors, no significant influence was found on the therapeutic effects induced by RPRIN, which may be due to the substantial structure of RPRIN preventing the MMP‐2‐induced digestion. However, the low pH conditions in the lysosomes and the abundant intracellular MMP‐2 synergistically destructed the nanoparticles and improved the release of miRNA, which was beneficial for lung cancer therapy.

Another remaining challenge is to protect the integrity and stability of miRNAs in the blood circulation. Naked miRNAs are easily digested for a short time by abundant nucleases in the blood, such as serum RNase, reducing the systemic half‐life.^36^ Additionally, the DLS analysis showed that the size and PDI of RPRIN were extremely stable in serum within 5 days, indicating the integrity and stability of RPRIN in the blood circulation.

RPRIN exhibits several benefits, such as prolonged circulation time, reduced toxicity, and immune evasion. Moreover, RPRIN exhibited higher transfection efficiencies than commercial transfection reagent Lipo3000. RPRIN showed tumor‐targeting and MMP2‐stimulated properties, enhancing the cellular uptake of miR‐148a‐3p in lung cancer cells. Both in vitro and in vivo experiments demonstrated that RPRIN was highly effective in inducing apoptosis in lung cancer cells and inhibited their growth and progression. We developed an effective and potent nanosystem based on tumor‐targeting and MMP2‐stimulated peptide binding miR‐148a‐3p for lung cancer therapy.

Our previous studies reveal a significant connection between the methylation status of miR‐148a‐3p and lung cancer metastasis. Therefore, it is necessary to evaluate the therapeutic effect of RPRIN on lung cancer patients with metastasis. In the current study, we only used subcutaneous tumor model for in vivo study. In the following research, in situ tumor model will be constructed to evaluate the role of RPRIN in the lung cancer metastatic progression.

## 4 CONCLUSION

In conclusion, we developed a mirrored RGD‐modified cationic peptide (RD24) as a miRNA vehicle. RD24 enabled to encapsulate miR‐148a‐3p efficiently and generated RPRIN, which effectively transduced miRNA into lung cancer cells. Furthermore, the pH and MMP‐2 responsiveness of RD24 induced a higher transfection rate than the commercial transfection reagent Lipo3000. Moreover, RPRIN showed extended circulation time, lower toxicity, and specificity to lung cancer cells. In vitro and in vivo experiments revealed that RPRIN effectively promoted lung cancer cell apoptosis and suppressed the development and progression of lung cancer. This type of mirrored RGD‐modified cationic peptide may be considered as a powerful tool for miRNA delivery in lung cancer treatment.

## 5 MATERIALS AND METHODS

### 5.1 Materials and reagents

The miR‐148a‐3p and Cy3‐miR‐148a‐3p were synthesized by Tsing Biotechnology Co., Ltd (Beijing, China). The polypeptide materials RRRRPLGLAGRRRR (RR14), RGDGSRRRRPLGLAGRRRR (RR19), RGDGSRRRRRRRRGSRGD (RR18), and RGDGSRRRRPLGLAGRRRRGSRGD (RD24) were synthesized by GL Biochem (Shanghai) Ltd (Shanghai, China). Human lung cancer cell lines A549, NCI‐H1299, NCI‐H460, and human lung epithelial cell line BEAS‐2B were from the Cell Bank of the Chinese Academy of Sciences. The αvβ3 integrin receptor antagonist (Cilengitide) was bought from FineTest (Wuhan, China). The fetal bovine serum (FBS) was from Inner Mongolia Opcel Biotechnology (Hohhot, China). Dulbecco's modified eagle medium (DMEM), phosphate buffer solution (PBS), RNase‐free water, and penicillin–streptomycin were the products of Gibco (Grand Island, NY, USA).

Male BALB/c nude mice (6 weeks) were obtained from Guangdong Medical Lab Animal Center. All the animal experiments were approved and supervised by the Animal Experiment Ethics Committee of Guangzhou Medical University (SYK2016‐0168, GY2021‐131).

### 5.2 Preparation and characterization of PIN, RPIN, and RPRIN nanoparticles

The PIN, RPIN, and RPRIN were formed by self‐assembly. RNase‐free water was used to dissolve the polypeptide materials and miR‐148a‐3p, for the preparation of 1 μg/μL of RR14, RR19, RD24, and miR‐148a‐3p solutions, respectively. Then 20 μL of the miR‐148a‐3p solution was added to 1 mL of RR14, RR19, and RD24 solutions with a weight ratio of 1/50, respectively. Afterwards, the mixture was vortexed for 30 s and then incubated at room temperature for 15 min to form nanoparticles.

The size and polydispersity index (PDI) of the nanoparticles were measured by Zeta Sizer (Malvern, Worcestershire, UK), and the morphological characteristics were captured by TEM (JEM‐1400 Plus, JEOL, Japan).

### 5.3 Agarose gel electrophoresis assay

Agarose gel electrophoresis assay was carried out for the analysis of residual miR‐148a‐3p. PIN, RPIN, and RPRIN were prepared with different weight ratios (RR14, RR19, or RD24:miR‐148a‐3p = 5:1, 10:1, 20:1, 30:1, 50:1, 60:1, 90:1), respectively. The samples were centrifuged at 4°C, 12,000 rpm for 30 min to separate the free miR‐148a‐3p and the loaded miR‐148a‐3p, then the free miR‐148a‐3p in the supernatant was analyzed by agarose gel electrophoresis to calculate the loading efficiency. The loading efficiency was calculated as the following formula: EE% = (*M*_total RNA_ − *M*_free RNA_)/*M*_total RNA_ × 100%.

### 5.4 Cellular uptake assay

NCI‐H1299 cells were incubated with PIN, RPIN, and RPRIN (Cy3‐miR‐148a‐3p equivalent to 4 μg/mL) for different time points (1, 4, 6, 8, and 10 h), respectively. For the dosage‐dependent assay, the NCI‐H1299 cells were treated with different concentrations of PIN, RPIN, and RPRIN (Cy3‐miR‐148a‐3p equivalent to 1, 2, 4, and 8 μg/mL) for 8 h, respectively. The qualitative and quantitative analyses were detected by confocal laser scanning microscope (CLSM, LSM 880, ZEISS, Germany) and flow cytometry (FACS, ImageStream Mark II, Merck, Germany).

### 5.5 Lysosomal escape assay

NCI‐H1299 cells treated with 4 μg/mL Cy3‐miR‐148a‐3p were stained with LysoTracker Green (Thermo Fisher Scientific Inc., USA) for 45 min. CLSM was used to test the distribution of lysosome and miRNA at different time points (1, 4, and 8 h), respectively.

### 5.6 Migration and invasion assays

The wound healing assay was used to detect the migration rates of nanoparticles. Transwell migration and invasion assays were performed using previously published methods^37^ to evaluate the effect of PBS, free miR‐148a‐3p, Lipo3000/miR‐148a‐3p, PIN, RPIN, and RPRIN on cell invasion and migration.

### 5.7 Cell viability assay

Cell viability was detected by CCK8 assay (Beyotime Biotechnology, China) based on the universal protocol.^38^ The ratio of cell viability was calculated as *A* treated/*A* control × 100%.

### 5.8 The transfection efficiency in vitro

NCI‐H1299 cells were treated with PBS, free miR‐148a‐3p, Lipo3000/miR‐148a‐3p, PIN, RPIN, and RPRIN (miR‐148a‐3p equivalent to 4 μg/mL), respectively. Following a 48‐h incubation period, the expression levels of miR‐148a‐3p were evaluated using the RT‐qPCR assay.^8^

### 5.9 In vivo tracking study

To detect the biodistribution of the miRNA‐loaded nanoparticles, PIN, RPIN, and RPRIN (equivalent to 20 μg Cy3‐miR‐148a‐3p per mouse) were administrated to the nude mice bearing NCI‐H1299 tumors by intravenous injection through the tail vein. The distribution of nanoparticles in mice was taken using IVIS Lumina III (PerkinElmer, USA) at 1, 4, 8, 12, 24, and 48 h post injection and analyzed by Living Image 4.5 software. Following in vivo tracking, the tumors and tissues of BALB/C nude mice were collected and analyzed using ex vivo imaging.

### 5.10 In vivo pharmacokinetics assay

We evaluated the circulation lifetime in the BALB/c nude mice. Briefly, free miR‐148a‐3p, PIN, RPIN, and RPRIN were administrated to the BALB/c nude mice by intravenous injection through tail vein (equivalent to 20 μg Cy3‐miR‐148a‐3p per mouse, *n* = 5). Then the whole‐blood samples of mice were collected for the detection of residual Cy3‐miR‐148a‐3p in circulation system at 1, 4, 8, 12, and 24 h after administration.

### 5.11 Nude mouse xenograft assay

The NCI‐H1299 cells (1 × 10^6^/100 μL) were subcutaneously injected into the right armpit of mice. After 14 days, the BALB/c nude mice bearing NCI‐H1299 tumors were randomly divided into five groups: Saline (*n* = 5); Free miR‐148a‐3p (treated with miR‐148a‐3p dissolved in saline, *n* = 5); PIN (treated with RR14/miR‐148a‐3p nanoparticles, *n* = 5); RPIN (treated with RR19/miR‐148a‐3p nanoparticles, *n* = 5); and RPRIN (treated with RD24/miR‐148a‐3p nanoparticles, *n* = 5). The treatments were conducted by intravenous injection through the tail vein every 2 days for 2 weeks. The bodyweight and tumor size were measured daily throughout the experiments. Tumor volume was calculated as (width^2^ × length)/2. Relative tumor volume (RTV)  =  *V*_t_/*V*_0_, where *V*_0_ is the tumor volume at day 0 of treatment and *V*_t_ is the tumor volume at day *t* of treatment. After sacrifice, the tumors were collected for TUNEL and EdU assay, H&E, and immunofluorescence staining. The major organs (livers, spleens, kidneys, hearts, and lungs) were harvested for H&E staining.

### 5.12 Immunofluorescence assay

For the immunofluorescence staining, the tumor sections were stained with primary antibody (anti‐E‐cadherin and anti‐N‐cadherin [ImmunoWay Biotechnology, Plano, USA]), secondary antibody (Servicebio, Catalog No. GB25301 [AF488] and GB21401 [Cy3], Wuhan, China). All the images were photographed by CLSM.

### 5.13 Statistical analysis

All the data were expressed as the mean ± standard deviation (SD). Comparisons were analyzed by the Student's *t*‐test. A two‐tailed *p* < 0.05 was considered significantly different.

## AUTHOR CONTRIBUTIONS

Lu Liang and Lingmin Zhang conceived and designed the experiments and contributed new reagents. Xiyong Yu supervised all the research. Wenyan Xu, Lingran Du, and Lina Yu performed the experiments. Huiyu Cen and Siran Wang analyzed the data. Zhixiong Ruan, Zhongxiao Lin, and Jishuo Chang wrote the original manuscript. Fangyu Lin, Xin Zhang, and Na Zhou revised the manuscript. All authors have approved the final version of the manuscript.

## CONFLICT OF INTEREST STATEMENT

The authors declare they have no conflicts of interest.

## ETHICS STATEMENT

All the animal experiments were approved and supervised by the Animal Experiment Ethics Committee of Guangzhou Medical University (SYK2016‐0168, GY2021‐131).

## Supporting information

Supporting Information

## Data Availability

All data are available from the corresponding authors upon request.
