# Supplementary material for: The mirrored cationic peptide as miRNA vehicle for efficient lung cancer therapy
Source: MedComm (2020). 2023 Jul 28;4(4):e273. doi: 10.1002/mco2.273 (PMC10382604; doi:10.1002/mco2.273)
Supplement: Supplementary file 1 — Supporting Information [file MCO2-4-e273-s001.docx]

**Supporting information**

**The mirrored RGD-modified cationic peptide as miRNA vehicle for efficient lung cancer therapy**

Wenyan Xu^1, #^, Lingran Du^1, #^, Lina Yu^1, 2 #^, Huiyu Cen^1^, Fangyu Lin^4^, Siran Wang^1, 2^, Zhixiong Ruan^1^, Zhongxiao Lin^1, 3^, Xin Zhang^3^, Na Zhou^3^, Jishuo Chang^1^, Xiyong Yu^1,^*, Lingmin Zhang^1,^ *, Lu Liang^1,^ *

^1^Guangzhou Municipal and Guangdong Provincial Key Laboratory of Molecular Target & Clinical Pharmacology, the State & NMPA Key Laboratory of Respiratory Disease, School of Pharmaceutical Sciences & The Fifth Affiliated Hospital, Guangzhou Medical University, Guangzhou, 511436, PR China

^2^Department of Preventive Dentistry, Affiliated Stomatology Hospital of Guangzhou Medical University, Guangdong Engineering Research Center of Oral Restoration and Reconstruction, Guangzhou Key Laboratory of Basic and Applied Research of Oral Regenerative Medicine, Guangzhou, 510182, PR China

^3^State Key Laboratory of Quality Research in Chinese Medicine, Macau University of Science and Technology, Avenida Wailong, Taipa, Macau, PR China

^4^Department of Ophthalmology, Emory University, B5500 Clinic B, 1365B Clifton Road NE, Atlanta, GA 30322, USA

^#^These authors contributed equally to this work.

*Correspondence to: Xiyong Yu, Lingmin Zhang, and Lu Liang

E-mails: [yuxycn@aliyun.com](mailto:yuxycn@aliyun.com), zhanglm@gzhmu.edu.cn and [lliangaa@gzhmu.edu.cn](mailto:lliangaa@gzhmu.edu.cn)

**Table S1 The detailed sequences and abbreviations of nanoparticles.**

| Abbreviations | Full name |
| --- | --- |
| RR14 | RRRRPLGLAGRRRR |
| RR19 | RGDGSRRRRPLGLAGRRRR |
| RD24 | RGDGSRRRRPLGLAGRRRRGSRGD |
| RR18 | RGDGSRRRRRRRRGSRGD |
| PIN | RR14/miR-148a-3p nanoparticles |
| RPIN | RR19/miR-148a-3p nanoparticles |
| RPRIN | RD24/miR-148a-3p nanoparticles |
| RRIN | RR18/miR-148a-3p nanoparticles |


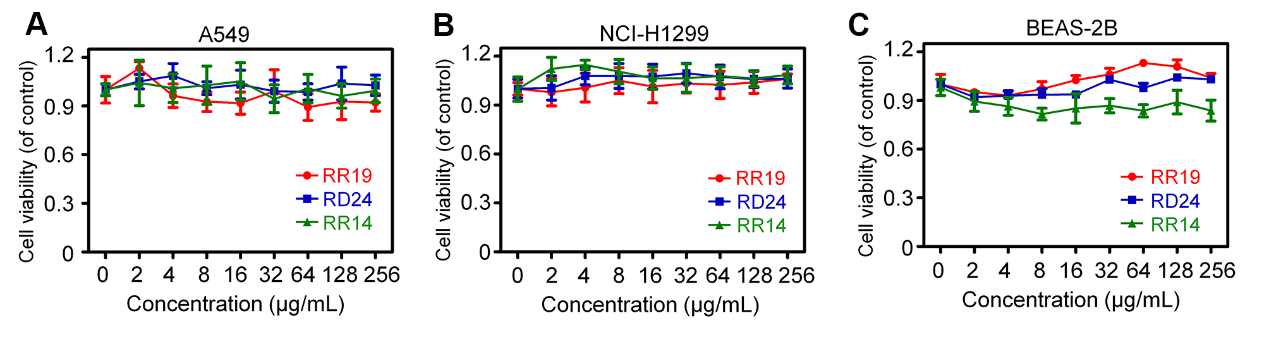


**Figure S1. Cell viability detection of A549, NCI-H1299 and BEAS-2B cells following treatment with RR14, RR19 and RD24.**


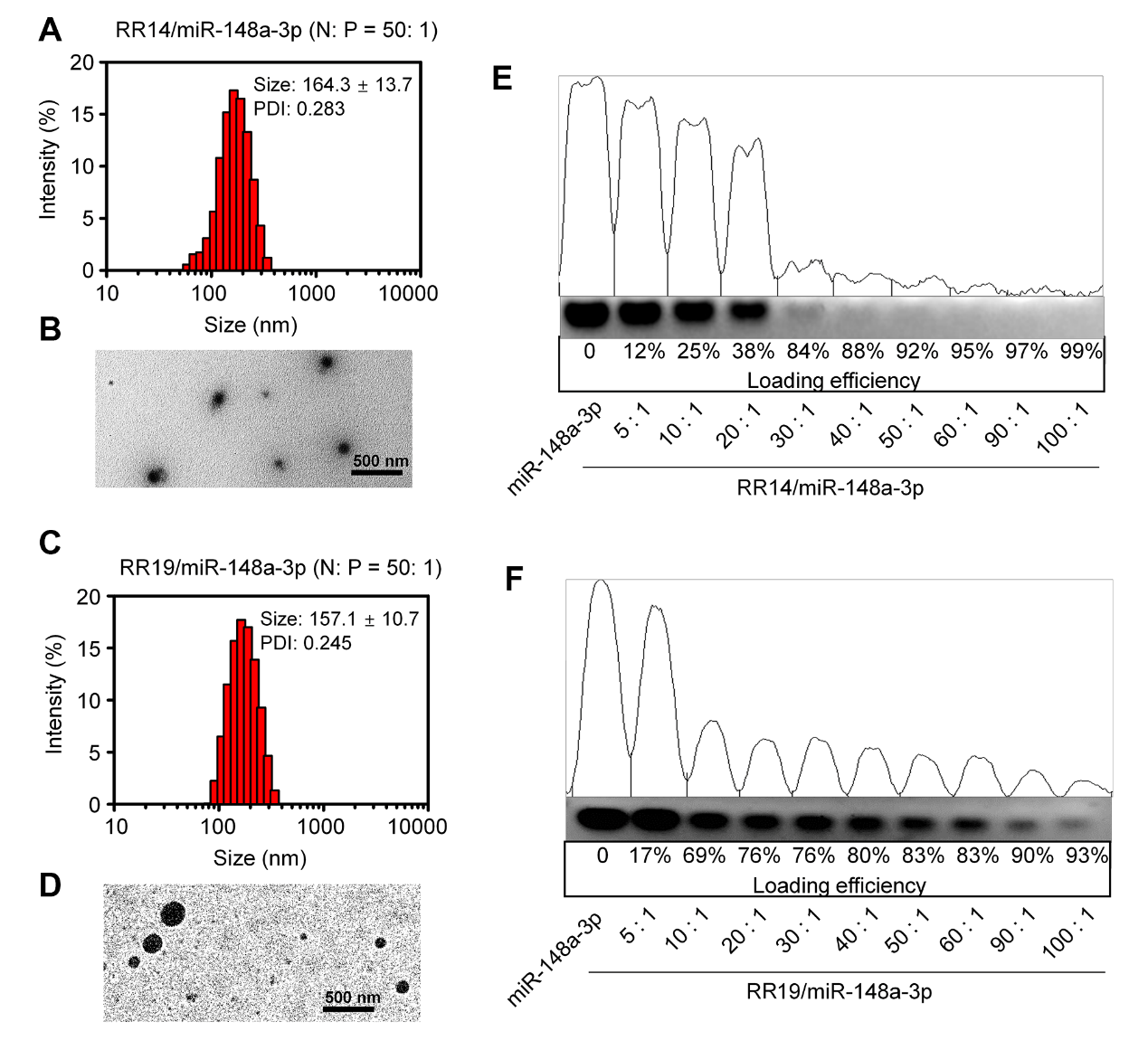


**Figure S2. The properties of RR14/miR-148a-3p (PIN) and RR19/miR-148a-3p (RPIN).** (A) The size distribution and (B) TEM image analysis of PIN. (C) The size distribution and (D) TEM image analysis of RPIN. (E) The loading efficiency of RR14 to miR-148a-3p. (F) The loading efficiency of RR19 to miR-148a-3p.


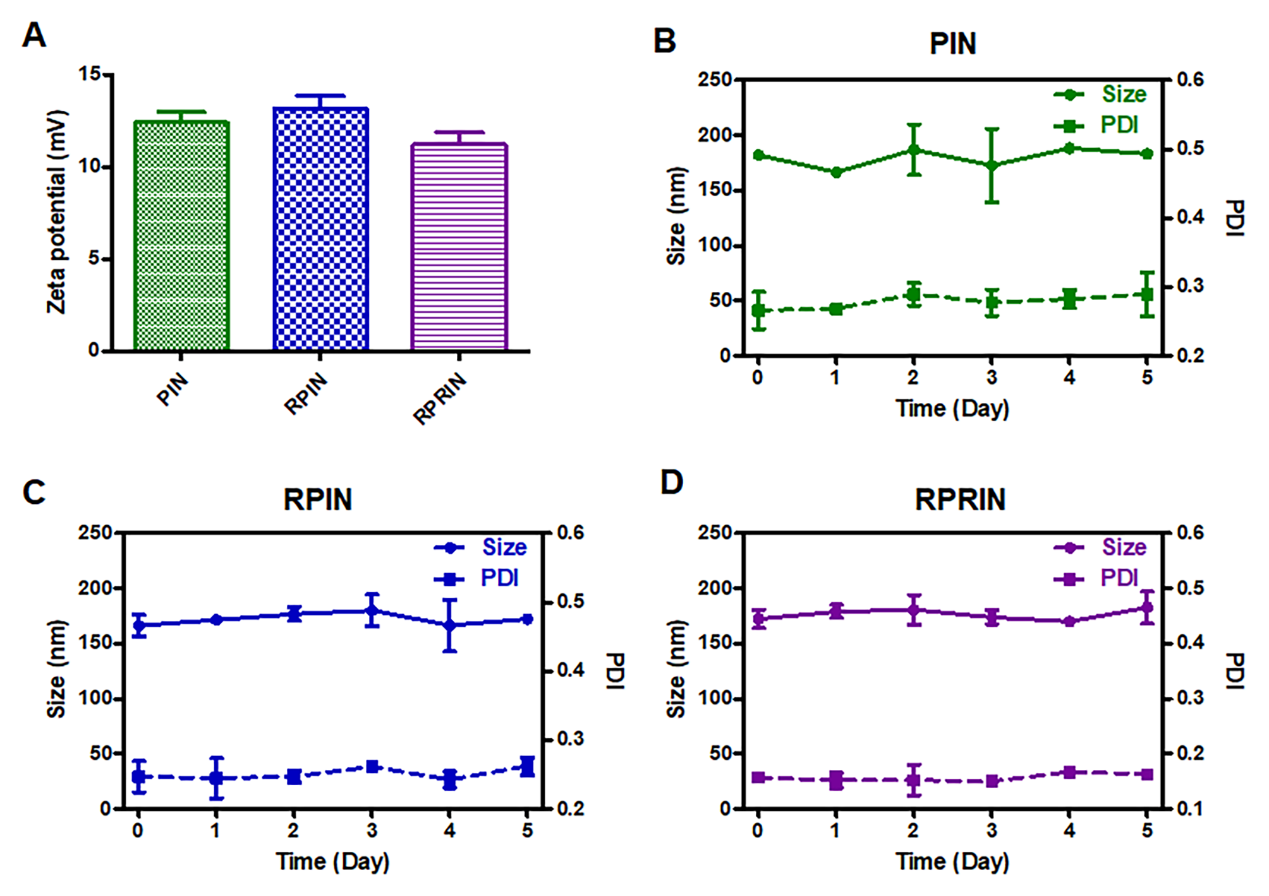
 **Figure S3. The physicochemical characterization of PIN, RPIN and RPRIN.** A) The zeta potentials of PIN, RPIN and RPRIN. B, C and D) The size and PDI characteristics of PIN, RPIN and RPRIN in serum within 5 days.


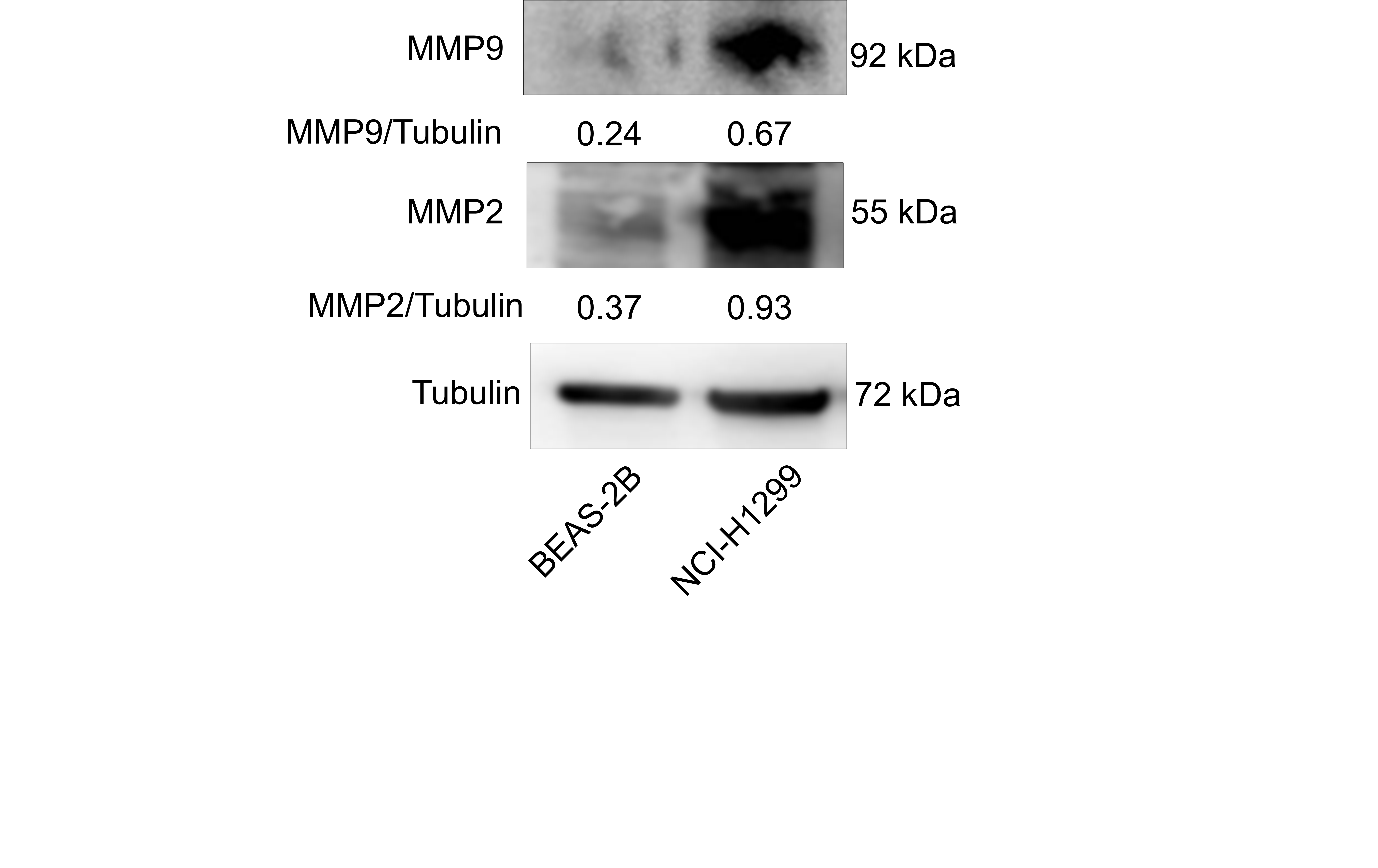


**Figure S4. Total protein of human bronchial epithelial cells (BEAS-2B) and lung adenocarcinoma cells (NCI-H1299) were harvested using RIPA lysis buffer, and the MMP-2 and MMP-9 levels were analyzed by western blot, respectively.**

**
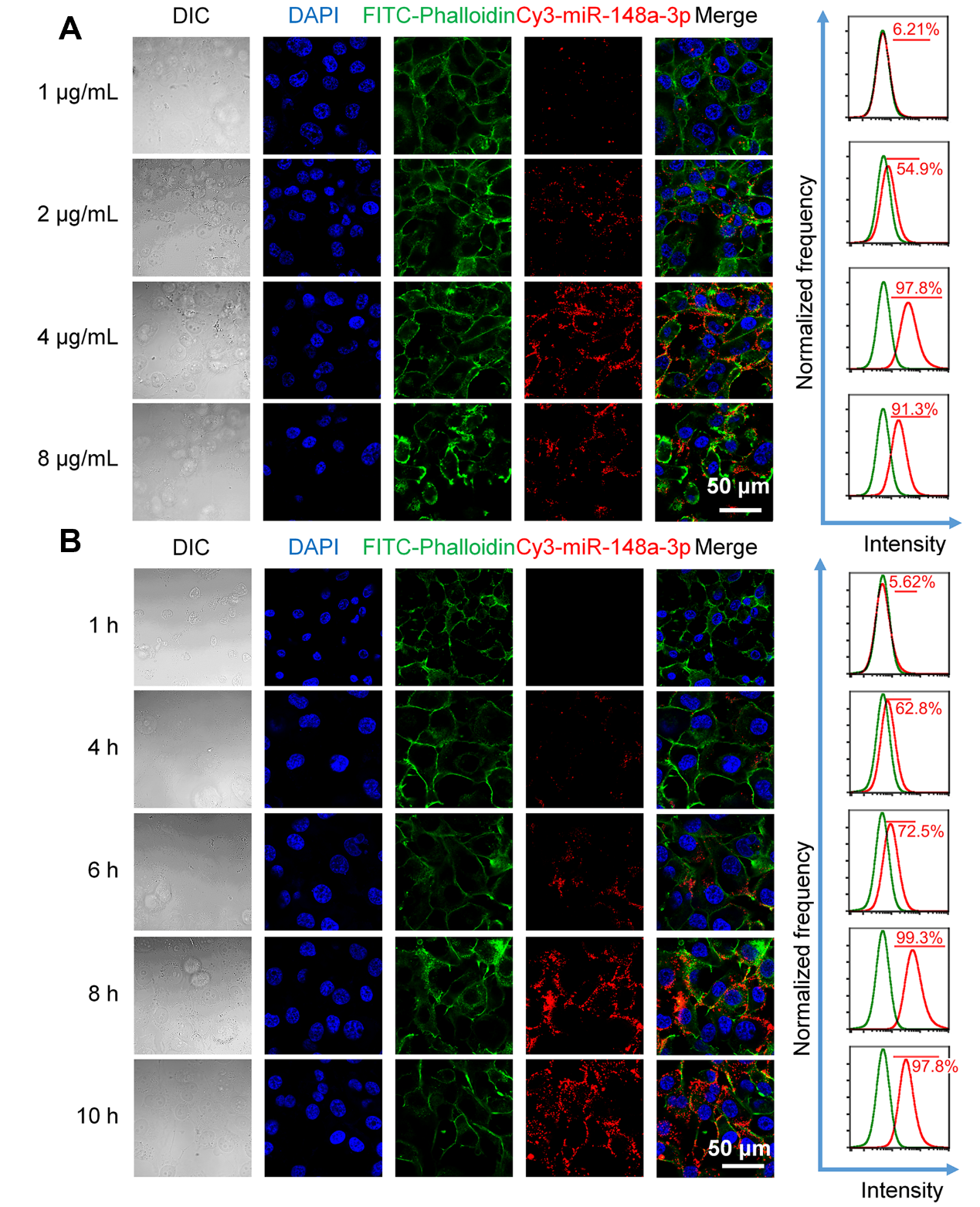
**

**Figure S5. The evaluation of cellular uptake** **in a time- and dose-dependent manner.** (A) The cellular uptake of RPRIN in a dose-dependent manner. NCI-H1299 cells were transfected with various concentrations of RPRIN (Cy3-miR-148a-3p equivalent to 1, 2, 4, and 8 μg/mL) for 8 h, respectively. Scale bar, 50 μm. (B) The cellular uptake of RPRIN in a time-dependent manner. NCI-H1299 cells were transfected with RPRIN (Cy3-miR-148a-3p equivalent to 4 μg/mL) for 1, 4, 6, 8, and 10 h, respectively. Scale bar, 50 μm.


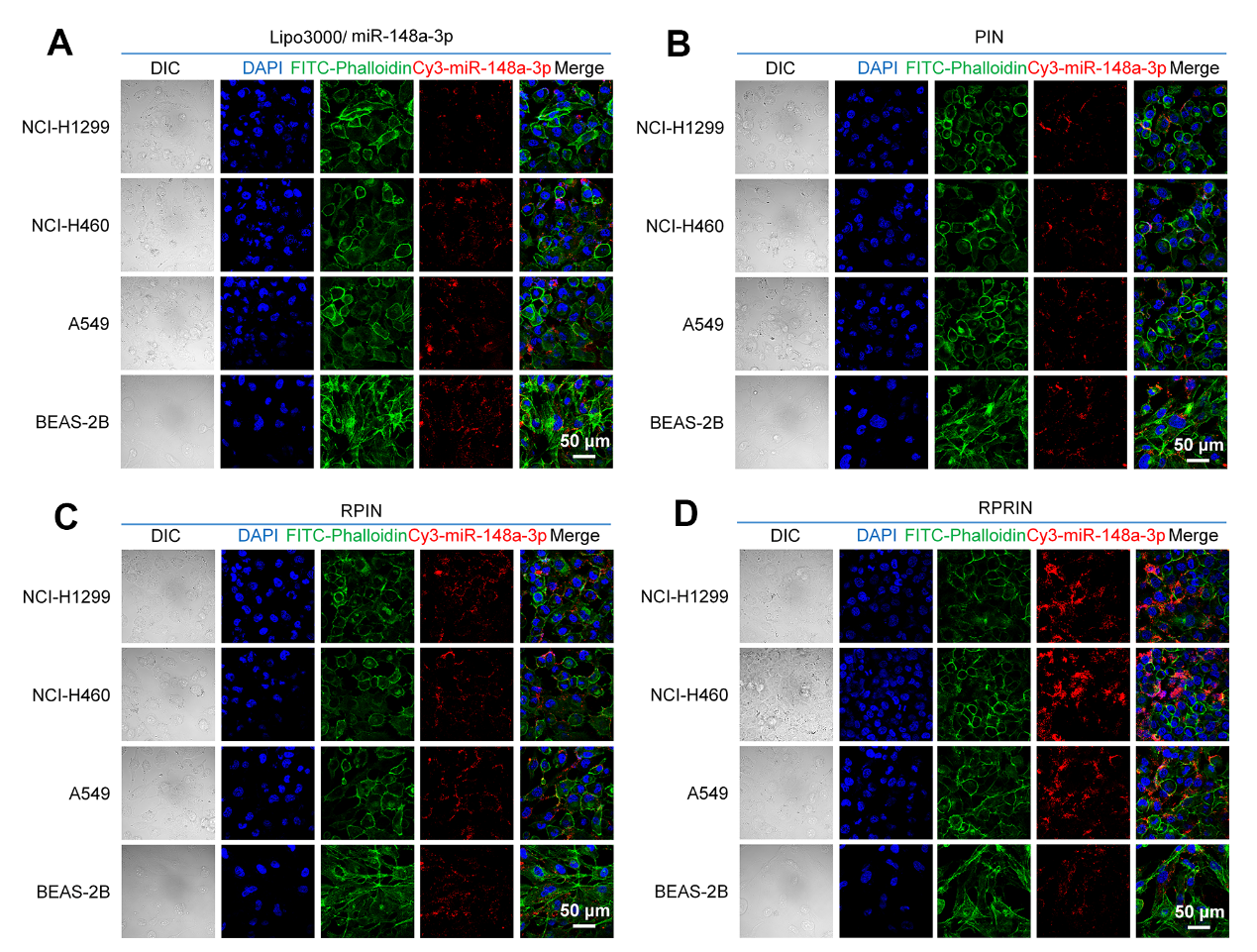


**Figure S6. The cellular uptake in different conditions by confocal laser scanning microscope.** (A) The cellular uptake of Lipo3000/miR-148a-3p containing 4 μg/mL miR-148a-3p in different cell lines (NCI-H1299, NCI-H460, A549 and BEAS-2B). (B) The cellular uptake of PIN containing 4 μg/mL miR-148a-3p in different cell lines (NCI-H1299, NCI-H460, A549 and BEAS-2B). (C) The cellular uptake of RPIN containing 4 μg/mL miR-148a-3p in different cell lines (NCI-H1299, NCI-H460, A549 and BEAS-2B). (D) The cellular uptake of RPRIN containing 4 μg/mL miR-148a-3p in different cell lines (NCI-H1299, NCI-H460, A549 and BEAS-2B).


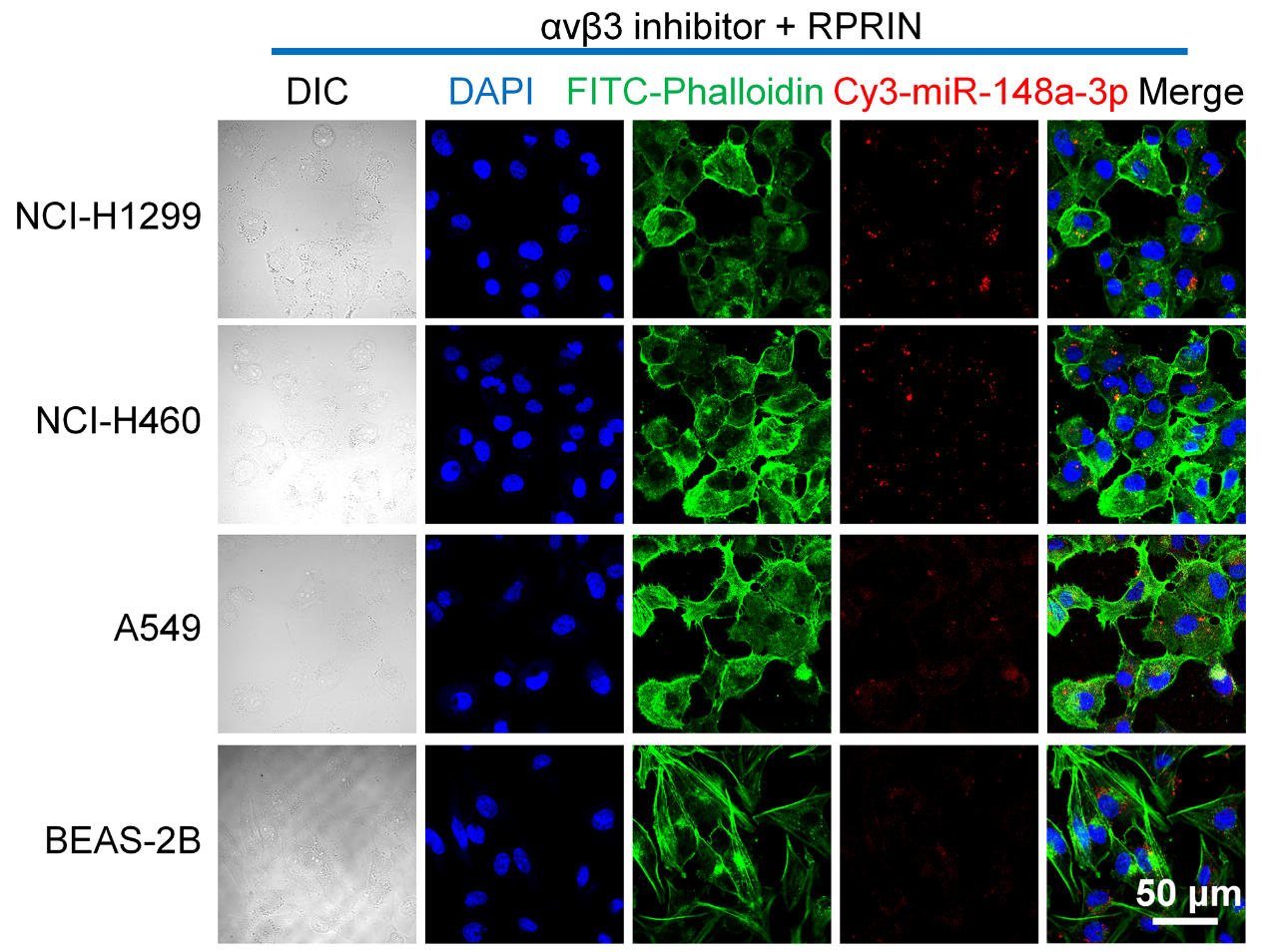


**Figure S7. The cellular uptake of RPRIN containing 4 μg/mL miR-148a-3p in different cell lines (NCI-H1299, NCI-H460, A549 and BEAS-2B) after treatment with αvβ3 inhibitor.**


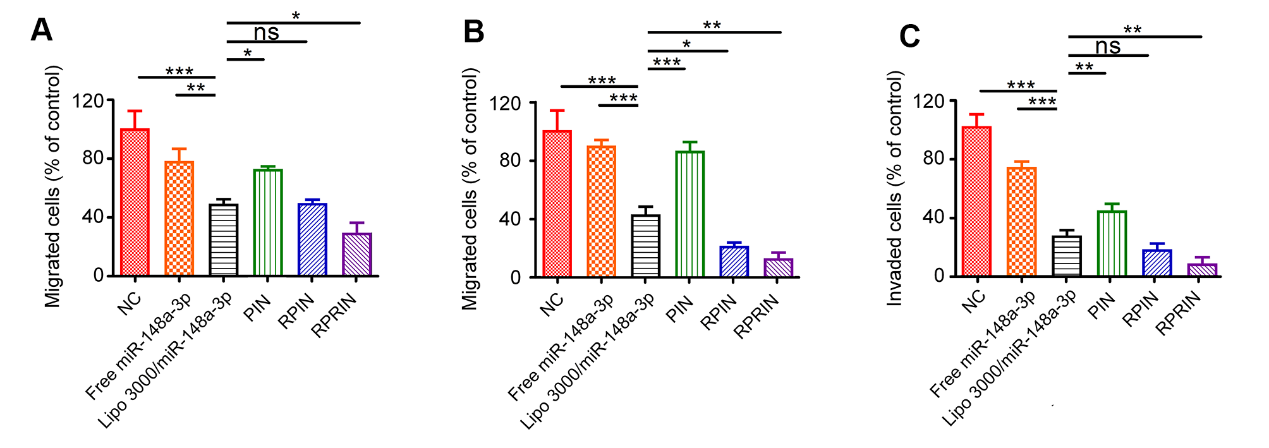


**Figure S8. Quantitative analysis of** **the effect of nanoparticles on cell migration and invasion.** (A) Wound healing assay measured cell migration. (B) Transwell assay measured cell migration. (C) Transwell assay measured cell invasion. *, *P* < 0.05; **, *P* < 0.01; ***, *P* < 0.001.


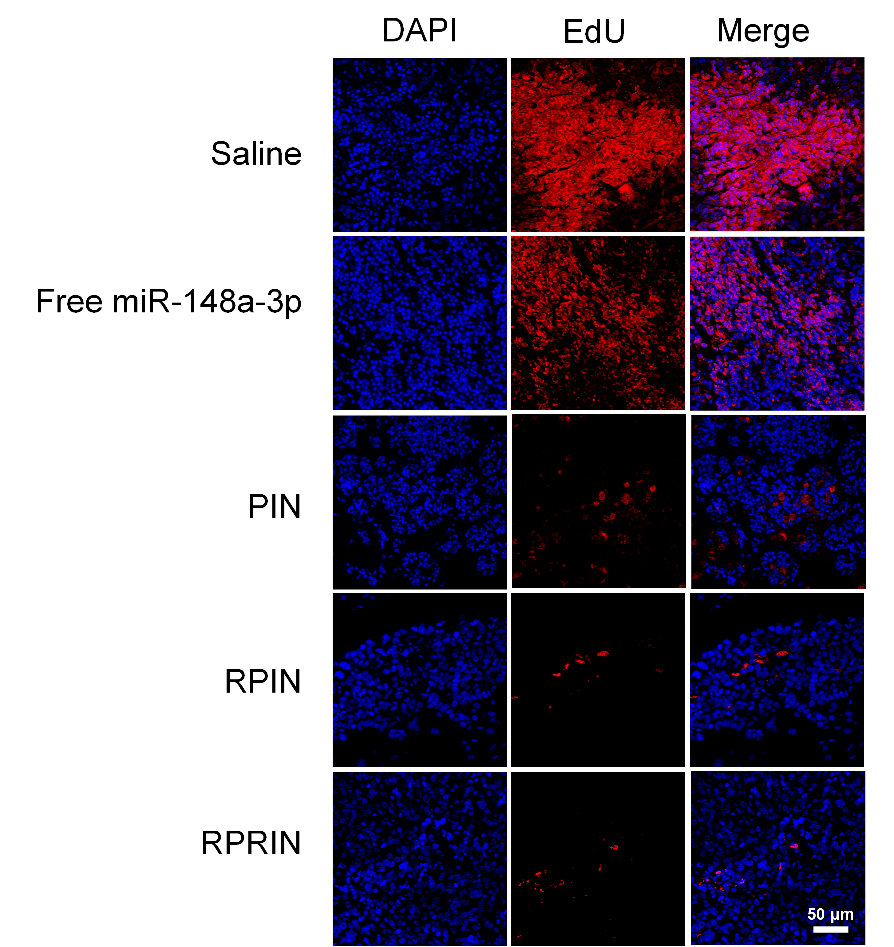


**Figure S9.** **EdU staining in the tumor tissues.**


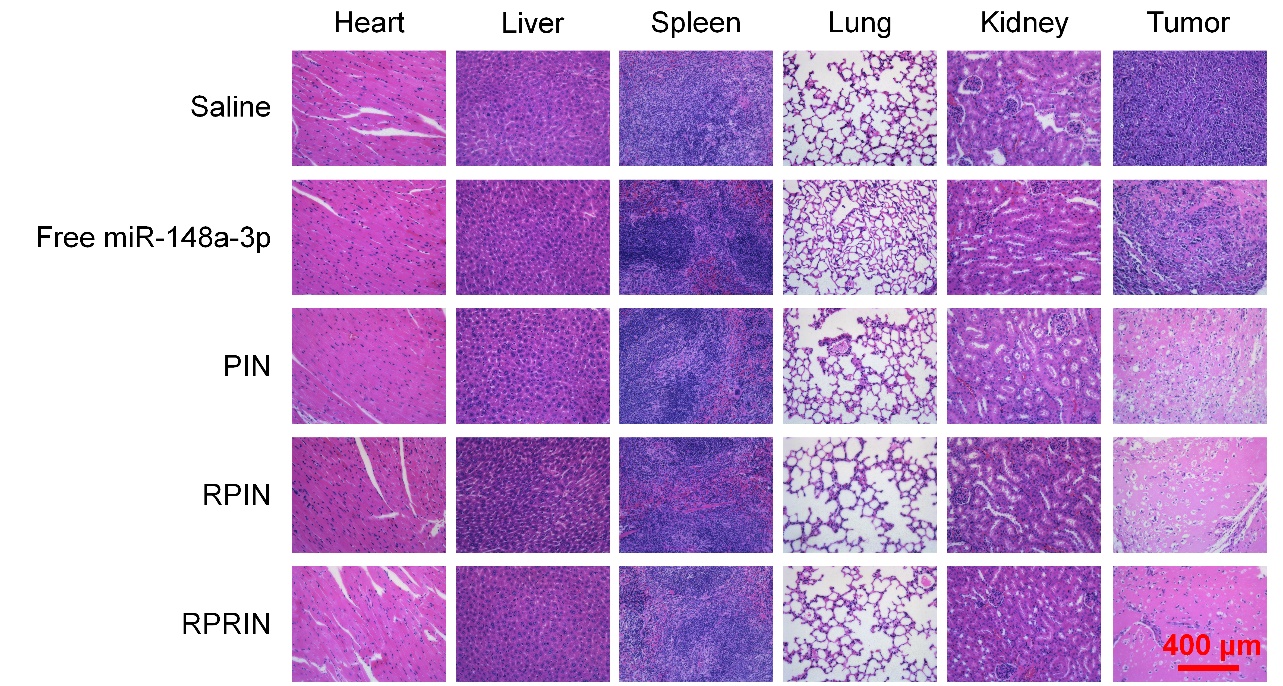


**Figure S10. Representative H&E-stained slices of major organs and tumors.**
